# SAMMD: *Staphylococcus aureus *Microarray Meta-Database

**DOI:** 10.1186/1471-2164-8-351

**Published:** 2007-10-02

**Authors:** Vijayaraj Nagarajan, Mohamed O Elasri

**Affiliations:** 1 Department of Biological Sciences; The University of Southern Mississippi, MS, 39406, USA

## Abstract

**Background:**

*Staphylococcus aureus *is an important human pathogen, causing a wide variety of diseases ranging from superficial skin infections to severe life threatening infections. *S. aureus *is one of the leading causes of nosocomial infections. Its ability to resist multiple antibiotics poses a growing public health problem. In order to understand the mechanism of pathogenesis of *S. aureus*, several global expression profiles have been developed. These transcriptional profiles included regulatory mutants of *S. aureus *and growth of wild type under different growth conditions. The abundance of these profiles has generated a large amount of data without a uniform annotation system to comprehensively examine them. We report the development of the *Staphylococcus aureus *Microarray meta-database (SAMMD) which includes data from all the published transcriptional profiles. SAMMD is a web-accessible database that helps users to perform a variety of analysis against and within the existing transcriptional profiles.

**Description:**

SAMMD is a relational database that uses MySQL as the back end and PHP/JavaScript/DHTML as the front end. The database is normalized and consists of five tables, which holds information about gene annotations, regulated gene lists, experimental details, references, and other details. SAMMD data is collected from the peer-reviewed published articles. Data extraction and conversion was done using perl scripts while data entry was done through phpMyAdmin tool. The database is accessible via a web interface that contains several features such as a simple search by ORF ID, gene name, gene product name, advanced search using gene lists, comparing among datasets, browsing, downloading, statistics, and help. The database is licensed under General Public License (GPL).

**Conclusion:**

SAMMD is hosted and available at . Currently there are over 9500 entries for regulated genes, from 67 microarray experiments. SAMMD will help staphylococcal scientists to analyze their expression data and understand it at global level. It will also allow scientists to compare and contrast their transcriptome to that of the other published transcriptomes.

## Background

*Staphylococcus aureus *is an important human pathogen, causing diseases ranging from superficial skin infections to severe life threatening infections. *S. aureus *is the foremost cause of nosocomial infections. *S. aureus *is also posing serious threats because of its ability to acquire multiple antibiotic resistances. Microarray studies enable the analysis of the pathogens response at a global level. There have been several studies carried out on the global expression profiles of *S. aureus *in response to different effectors like vancomycin [1], mild acid [2], stress [3] etc. There are also several transcriptional profiles of regulatory genes like *sigB *[4], *sarA *[5], *mgrA *[6] etc. To date there are about 30 published journal articles that contain about 67 microarray experiments in *S. aureus*. The use of this large amount of expression data is limited by the fact that it is not located in a centralized source. In addition, those data that have been deposited in the public databases are difficult to use for direct comparisons to data generated by researchers. We have addressed this issue by building a *Staphylococcus aureus *microarray meta-database (SAMMD) which contains all the published microarray data generated for *S. aureus*. SAMMD is a web accessible database that allows users to mine for information about a single or several genes. SAMMD can also be used to compare a whole transcriptome to published data.

### Need for the database

Databases are increasingly useful in biology as huge amount of data is generated by high throughput techniques such as Microarray technology. Computational tools are essential to analyse the vast mines of archived data and generate biological information. Scientists are encouraged to deposit published data in public databases such as the National Centre for Biotechnology Information – Gene Expression Omnibus (NCBI-GEO) [7] or European Bioinformatics Institute (EBI) Array Express [8], or Stanford Microarray Database (SMD) [9]. To date, most groups however, have not complied resulting in a large amount of published DNA microarray data that is inaccessible for further analysis by other scientists. This makes it difficult to manipulate data or make comparisons with other experiments.

Even when raw data is available online, the lack of computational tools and expertise as well as the difference in platforms used to generate the data makes difficult to take full advantage of these resources. SAMMD addresses these issues by providing a central location for *S. aureus *microarray data. SAMMD was designed to allow users to quickly and easily mine the vast and growing collection of *S. aureus *transcriptomic data across different platforms. The search functions in SAMMD allow in depth analysis for the expression of one or collections of genes. SAMMD is a valuable tool for understanding the molecular mechanisms of pathogenesis in *S. aureus*.

### Forerunners and competitors

SAMMD is the first database that contains all the transcriptomic data for *S. aureus*. Databases devoted to other organisms have been developed. For instance, The Saccharomyces Genome Database (SGD) [10] is devoted to the yeast, *Saccharomyces cerevisiae*. SGD is a comprehensive genome database, which also contains information about several yeast Microarray experiments. Two other similar databases are devoted to *E. coli *gene expression [11], and to the human microarray data (LOLA) [12].

### Potential value

SAMMD is a highly valuable tool for staphylococcal research. SAMMD is useful to study a single gene, several genes, or a genome-wide transcriptome. SAMMD can also be used to gain insights about mutational status using transcriptomic data. Since SAMMD will be updated constantly as new transcriptomes are published, its utility and value will continue to grow.

## Construction and content

### Database schema

SAMMD is a relational database consisting of five tables (Database schema is presented in the SAMMD help page). The "annotation" table includes information about ORF IDs from six different strains of *S. aureus *(N315, COL, Mu50, MW2, MRSA252 and MSSA476), obtained from the primary source (National Centre for Biotechnology Information). The "experiment" table includes information about the Microarray experiments including the regulator or growth condition, number of replicates, strains, array platform, data analysis software, fold change cut-off value, and Pubmed ID (PMID). In addition, the experiment table will indicate whether RNA stabilising agents were used and if the raw datasets are available. The "reference" table contains references to published journal articles from which the data was extracted. The "regulated" table contains the list of differentially expressed genes (represented from the all the above mentioned strains of *S. aureus*), the effect of the mutation or growth condition on each gene (up or down). A table labelled "others" consists of information about non-transcriptomic DNA Microarray experiments such as genome comparisons. Corresponding primary and foreign keys are used to link the tables used in SAMMD.

### Implementation

SAMMD was implemented with MySQL 4.1.10 as the back end. PHP 4.3.9, JavaScript and DHTML were used to develop the front end user interface (Implementation scheme is presented in the SAMMD help page). phpMyAdmin 2.9.2 was used as the database administration tool as well as for data entry. The entire project is hosted as a bioinformatics.org project. The user entered query is sent to a PHP script through an interactive form. The PHP script sends the queries to MySQL database. The PHP script then retrieves and displays results, with hyperlinks to additional information. For every hit that is returned, additional information is presented through a JavaScript pop up window that gets the data from another appropriate PHP script.

### Data sources and quality control

**Figure 1:**
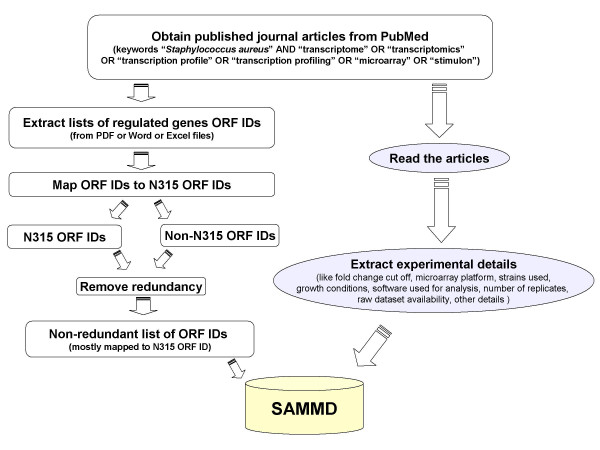
SAMMD Data Informatics.

Data for SAMMD is obtained by manually curating the published journal articles related to DNA Microarray studies in *S. aureus *(Figure 1). The following keywords were used to find appropriate articles in the PubMed database: "stimulon", "transcriptome", "transcriptomics", "transcription profile", "transcription profiling", "microarray". Each keyword was paired with the term "*Staphylococcus aureus*". We are using the same set of keywords to receive automatic email alerts from PubMed about the new *S. aureus *Microarray research articles, for the purpose of updating our database.

### Data extraction

Details about the experiments such as fold change cut off, Microarray platform, strains, etc. were obtained by carefully studying the journal articles. The related lists of regulated genes were extracted from either the journal article (from PDF files) or their respective supplemental files (Word or Excel files). The extracted lists of ORF IDs were mapped to N315 ORD IDs, using perl scripts, to enable comparison studies. The mapping scripts were written based on TIGR annotation files (*S. aureus *Version 6). Currently the mapping to N315 ORF ID is done for ORF IDs from 5 strains (MW2, COL, Mu50, MRSA252, and MSSA476). Genes that are not found in strain N315 are included in the database using their original name. The extracted data was entered in to the database using phpMyAdmin interface.

Quality of the data was checked at various points either by perl scripts or manual inspections. For instance, most of the *S. aureus *transcriptional experiments have few redundant genes from different strains represented in the same slide. These redundant genes are also reported as different entries in the list of regulated genes. In SAMMD, such duplicate entries were removed from the extracted and mapped gene lists. SAMMD has a feed back form, which the users could use to email the authors about any errors or other problems that they encounter.

## Utility and discussion

### User interface

The user interface was developed using PHP, JavaScript and DHTML. Users can browse through the transcriptomic content using the following categories: "transcriptomes of regulatory genes", "transcriptomes under different growth conditions", "transcriptomes of different strains", "transcriptomes published during a specific year", "transcriptomes published in a specific journal" and "transcriptomes in a specific reference".

**Figure 2:**
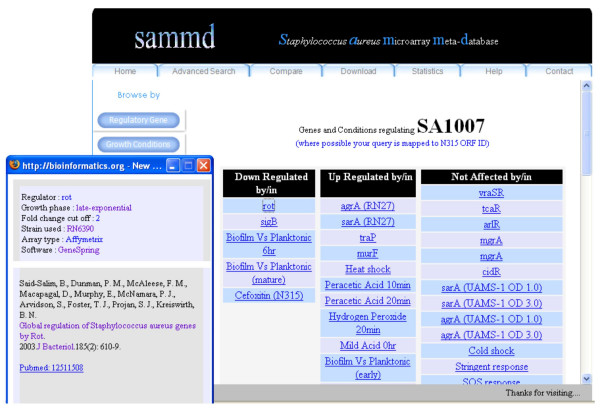
**Output of a simple search in SAMMD using an ORF ID**. ORF ID "SA1007" has been used as a query term in this search. All terms in the output are hyperlinked to further information.

**Figure 3:**
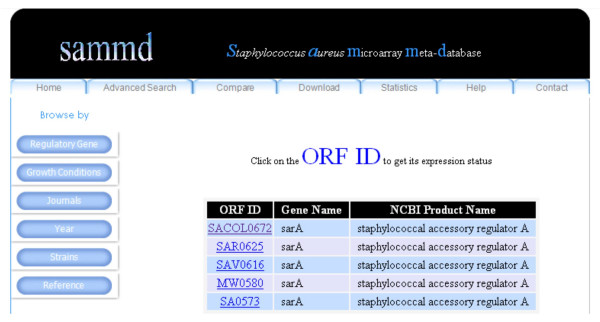
**Output of a simple search in SAMMD using a Gene name**. "*sarA*" has been used as a query term in this search. Clicking on the ORF ID would pull out its transcriptomic status in SAMMD, after mapping it to N315 ID.

A powerful search tool provided in the home page of the database provides users the ability to search for the transcriptomic status of a particular gene (Figure 2). Users can search using an ORF ID (e.g. SA1233) or a Gene name (e.g. *sar*A) or a gene product name (e.g. urease). The search by ORF ID option accepts ORF IDs from any of the following six strains of *S. aureus *(N315, MW2, Mu50, COL, MRSA252, MSSA476) as input. After execution of the search, the user is provided with an output that lists all of the experiments or transcriptomes where the query gene is found. The output also shows how the query gene was affected by the regulatory gene or growth conditions i.e. up regulated, down regulated or not affected. For a search using a Gene name, SAMMD accepts any standard *S. aureus *gene name and first returns a list of ORF IDs that have similar gene name (Figure 3) from the above mentioned strains of *S. aureus*. Clicking on a particular ORF ID shows the transcriptomic status of that particular ORF ID (usually mapped to N315 ID) in all different transcriptomes available in SAMMD.

We have also implemented a full text search against the "NCBI Product Name" column of the "annotation" table, under the search by "ORF Function" option. This lets users execute searches in SAMMD using key words. This full text search is Boolean operator enabled allowing users to add operators such as "AND", "OR", "NOT", "" and "*" to limit their search. The SAMMD help page contains more detailed help with examples about the usage of these Boolean operators.

**Figure 4:**
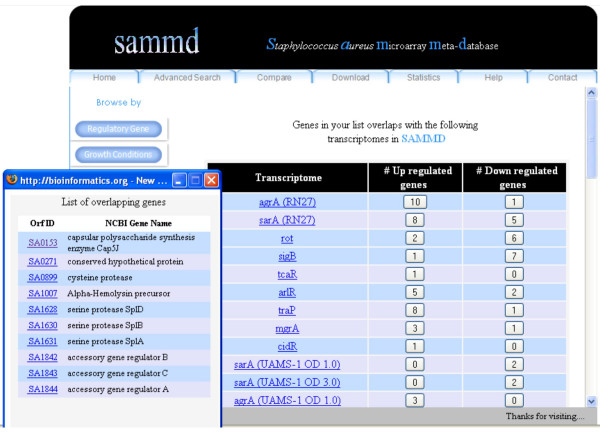
**Output of an advanced search in SAMMD using a list of ORF IDs**. Output shows the overlapping transcriptomes, with the corresponding list of genes. All terms in the output are hyperlinked to further information.

The advanced search lets the users to search SAMMD using a list of genes. Users can input a list of genes (any number of genes), as corresponding N315 ORF IDs. The output is the list of transcriptomes that has overlapping genes with that of the user entered gene list (Figure 4).

### Comparing datasets

**Figure 5:**
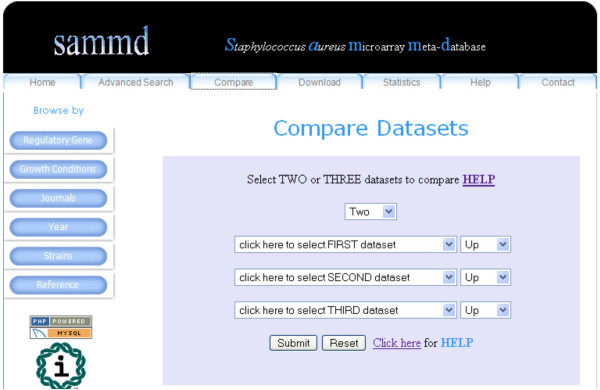
**Compare Datasets option**. Users can compare among different datasets using this option.

**Figure 6:**
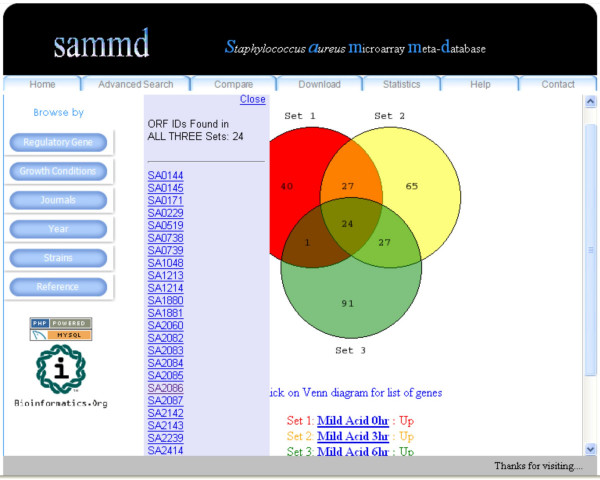
**Venn diagram showing the results of comparing three datasets**. Clicking on the numbers in the Venn diagram shows the corresponding list of genes in a pop up window.

SAMMD users can compare two or three datasets using the "Compare" menu option (Figure 5). Comparison between datasets is performed by set operations using PHP. The results are displayed in a Venn diagram, with the numbers in the diagram linked to the list of overlapping genes (Figure 6). The SAMMD help page shows an illustration of the dataset comparison option.

Options are also available for users to download relevant data from the database. Current statistics about the number of records in the database and a detailed help page with example gene lists and usage illustrations are also available. Contact information is also included for additional help with SAMMD and to receive comments and suggestions from users in order to improve the database.

### Intended use and benefits

SAMMD would be of immense use to scientists who are working on *S. aureus*. Knowing the transcriptional status of a particular gene from the literature might be a cumbersome task, because of the different semantics that are used to denote the genes from different strains. Manual searching for the transcriptional status of a particular gene becomes a laborious task, given the number of experiments and the huge number of regulated gene lists. SAMMD helps molecular microbiologists to over come these problems.

Comparative transcriptome analysis becomes conveniently possible with the help of SAMMD. Scientists who perform Microarray based experiments, could now easily compare their list of regulated genes (transcriptome) to that of the other transcriptomes in the database. They could also compare among the datasets that are already in SAMMD. This could help them to find out regulatory patterns and connections between their transcriptome and other transcriptomes, using the list of overlapping genes.

### Future developments

We are planning to incorporate a graphic module to represent the list of overlapping genes that is generated as a result of advanced search using a user entered list of genes.

## Conclusion

### Importance and relevance of the database

Microarray gene expression databases like NCBI-GEO and EBI-ArrayExpress hold the raw data only for a very few of the 30 published Microarray papers that are listed in SAMMD. The raw data that are published in these databases are not easily accessible for the use by biologists.

By developing SAMMD, we have addressed these issues. SAMMD is a searchable database of Microarray gene expression data of *S. aureus*. Such a database is valuable in staphylococcal research in light of increasing multiple-antibiotic resistance in *S. aureus*. SAMMD will allow scientists to study the role of individual genes in the context of global transcriptomes as well as enable comparison of new transcriptomes to published ones. SAMMD will facilitate understanding of the complex regulatory networks of *S. aureus*.

## Availability and requirements

The database is entirely based on open source concept and hence its usage is licensed under GNU General Public License (GPL). The database is available at the URL: 

SAMMD works best using the open source browsers like Firefox. JavaScript based pop-up windows are used to display some of the data in SAMMD, so the users should have JavaScript enabled their browsers for the full functionality of the database. Much of the data in the database could be directly downloaded from the database website. Any other file used in the development of the database would be provided up on request.

## Abbreviations

SAMMD: Staphylococcus aureus Microarray Meta-Database

ORF: Open Reading Frame

GPL: General Public License

NCBI: National Centre for Biotechnology Information

GEO: Gene Expression Omnibus

EBI: European Bioinformatics Institute

SGD: Saccharomyces Genome Database

SMD: Stanford Microarray Database

PMID: PubMed ID

## Competing interests

The author(s) declares that there are no competing interests.

## Authors' contributions

VN designed/developed the database, curated the data and drafted the manuscript. MOE directed the whole research and critically revised the manuscript. All the authors have read and approved the final version of the manuscript.
